# Dual Minimization of Spectrum Overlap for High-Sensitivity, High-Temperature Sensing

**DOI:** 10.3390/s26010126

**Published:** 2025-12-24

**Authors:** Xiaoheng Xu, Ke Shen, Xuankang Zhang, Yujian Liu, Yan Qian, Quli Fan

**Affiliations:** State Key Laboratory of Flexible Electronics (LoFE), Institute of Advanced Materials (IAM), Nanjing University of Posts & Telecommunications, Nanjing 210023, China; b22060428@njupt.edu.cn (X.X.); 1022233817@njupt.edu.cn (K.S.); 1223066432@njupt.edu.cn (X.Z.); 2022060715@njupt.edu.cn (Y.L.)

**Keywords:** film thermometer, spectrum overlap, heat resistant organic luminophores, high temperature sensing, anti-counterfeiting

## Abstract

**Highlights:**

**What are the main findings?**

- Dually minimizing spectral overlap of both energy transfer and emission enables high-sensitivity ratiometric thermosensing.
- Enlarging the emission separation between dual emitters proves more decisive for enhancing sensitivity than suppressing FRET or enhancing thermal decay contrast.

**What are the implications of the main findings?**

- Providing new insights for understanding underlying mechanism in high-sensitivity thermometry.
- Providing a new design strategy for fabrication high-performance thermometers.

**Abstract:**

Minimizing the spectrum overlaps of energy transfer (ET) is necessary but not sufficient for achieving high-sensitivity film thermosensing. Herein we have designed two blue emitters of DBA-BPAc and Z-DBABH exhibiting blue and bluish-green emissions, respectively, to hybridize with the red-emitting Ir(MDQ)_2_(acac). Compared with Z-DBABH, DBA-BPAc shows a larger spectrum overlap of ET and a relatively smaller discrepancy in fluorescence thermal decay, while its emission spectrum displays a much smaller overlap with that of Ir(MDQ)_2_(acac). The dual minimization of spectrum overlap of ET and emissions results in its superior ratiometric film thermosensing of the DBA-BPAc film in wide-range and high-temperature regions. The DBA-BPAc/Ir(MDQ)_2_(acac) film exhibits a maximum relative sensitivity (S_r_) of 3.36% °C^−1^ at 166 °C, exceeding 0.43% °C^−1^ in 50–265 °C. In comparison, the Z-DBABH/Ir(MDQ)_2_(acac) system displays a reliable but relatively lower performance, with a maximum S_r_ of 1.92% °C^−1^ (at 300 °C). The temperature resolution remains below 2.06 °C throughout the entire temperature range (20–300 °C), achieving a best value of 0.60 °C at 180 °C. Notably, both films display distinct naked-eye color transitions with temperature changes, enabling multi-level anti-counterfeiting applications. This work provides new insights for designing high-performance thermometers.

## 1. Introduction

Temperature is one of the most fundamental physical parameters in both scientific research and technological applications and precise temperature monitoring is crucial for modern industries including e-skin electronics, climate monitoring, and marine research [1,2,3,4,5,6]. Among various temperature-measuring methods, luminescent thermometers have attracted considerable attention owing to their non-invasive detection, fast response, excellent spatial resolution, capability for monitoring moving objects, and immunity to electrical and magnetic interference [7,8,9,10,11,12]. Luminescent thermometers operate by monitoring temperature-dependent photophysical parameters such as emission intensity, ratiometric emission intensity, and emission lifetime [13,14,15,16,17,18,19,20,21]. Among these approaches, intensity-based thermometry is highly sensitive to environmental interference, such as luminophore concentration, fluctuations in excitation source or detector and background luminescence, and often suffers from poor stability and repeatability, whereas lifetime-based thermometry requires high-cost and time-consumable instruments. In contrast, ratiometric thermometers, which rely on the temperature-dependent intensity ratios of two distinct emissions, provide more reliable and accurate measurements owing to their self-referencing characteristics, with no requirement of expensive instrument [22,23].

Ratiometric thermometers are typically based on inorganic metal complexes, metal–organic frameworks (MOFs) or organic luminophores, etc. [24,25,26,27,28,29,30]. Among them, organic fluorophores have garnered widespread attention as potential candidates due to their intrinsic flexibility and ease of molecular tailoring [31,32,33]. However, during heating, organic emitters are prone to undergo significant non-radiative deactivation, leading to substantial thermal quenching and a prominent decrease in detection sensitivity in the high-temperature region. Therefore, the development of heat-resistant organic luminophores is critical for achieving ratiometric thermometers with high sensitivity over a wide temperature window.

Generally, a ratiometric film thermometer is fabricated by using a heat-resistant emitter with a fast thermal quenching emitter. This high contrast in their emission intensity is vital for achieving high temperature sensitivity, but is often diminished by unavoidable energy transfer between two emitters. In our previous work, we proposed several strategies to suppress energy transfer in two emitters exhibiting markedly different thermal responses for realizing high-sensitivity ratiometric film thermometers for high-temperature and wide-range thermosensing [34,35,36,37,38,39,40]. Minimizing the spectral overlap between the donor emission and acceptor absorption [34,35,36] or enlarging the donor–acceptor distance by phase separation [37] can effectively inhibit energy transfer and largely preserves their independent thermal responses. In this work, by delicately designing two hybrid ratiometric emitter systems, it has been demonstrated that in addition to reducing the energy transfer spectrum overlap and large discrepancy in thermal responsiveness in luminescence intensity, the spectral separation between the donor and acceptor emissions plays a more vital role in obtaining high sensitivity in ratiometric thermometry. This work provides new insights for designing low-cost and high-sensitivity ratiometric film thermometers for wide-range thermal sensing, including high-temperature regions.

## 2. Materials and Methods

All films were prepared using PMMA as the polymer matrix at a weight ratio of 1 wt%. Mixed films were prepared by drop-casting mixed solutions with varying ratios onto quartz surfaces. UV-vis absorption and PL spectra were recorded using a Hitachi UV-3010 and Hitachi F-7000 spectrophotometer from HITACHI (Tokyo, Japan), respectively. Lifetime decay spectra were measured using an Edinburgh FLSP920 lifetime spectrometer from Edinburgh Instruments Ltd. (Edinburgh, UK). A high-temperature fluorescence controller, TAP-02, from Tianjin Dongfang Kejie Technology Co., Ltd. (Tianjin, China) was used for heating experiments.

**Synthesis of DBA-BPAc.** A mixture of p-bromoacetophenone (2.00 g, 10.05 mmol), BPABT (5.26 g, 10.05 mmol), PdCl_2_(dppf) (0.294 g, 401.92 μmol), and K_2_CO_3_ (5.55 g, 40.19 mmol) was added to a 250 mL Schlenk tube. After purging 12 mL of distilled water and 40 mL of ethanol with nitrogen, the solvents were added to dissolve the reactants. The reaction mixture was stirred at 55 °C for 3 h in inert atmosphere. Upon completion, the reaction mixture was cooled to room temperature and extracted with dichloromethane. The combined organic layers were dried over anhydrous Na_2_SO_4_, filtered, and concentrated under reduced pressure to give a crude product. The residue was purified by silica gel column chromatography using petroleum ether/dichloromethane (*v*/*v* = 1:1) as the eluent, affording a bright yellow solid in 74% yield. ^1^H NMR (400 MHz, CDCl_3_) δ 8.03 (d, J = 8.2 Hz, 2H), 7.69 (d, J = 8.2 Hz, 2H), 7.64–7.51 (m, 10H), 7.44 (t, J = 7.6 Hz, 4H), 7.33 (t, J = 7.4 Hz, 2H), 7.24 (s, 3H), 2.64 (s, 3H), 1.54 (s, 3H). MALDI-TOF *m*/*z*: 515.22 [M]^+^. HRMS (EI) *m*/*z*: 515.2240 [M]^+^. Anal. calcd for C_38_H_29_NO (515.2249).

**Synthesis of Z-DBABH.** Z-DBABH was prepared according to a procedure similar to that of DBA-BPAc, by using a dibromo compound of (Z)-1,3-bis(4-bromophenyl)-3-hydroxyprop-2-en-1-one (ZPhBr), giving a bright yellow solid in 40% yield. ^1^H NMR (400 MHz, CDCl_3_) δ 8.08 (d, J = 8.1 Hz, 4H), 7.73 (d, J = 8.1 Hz, 4H), 7.64–7.52 (m, 20H), 7.44 (t, J = 7.6 Hz, 8H), 7.37–7.23 (m, 13H), 6.95 (s, 1H), 1.26 (d, J = 6.1 Hz, 2H). MALDI-TOF *m*/*z*: 1014.42 [M]^+^. HRMS (EI) *m*/*z*: 1014.4160 [M]^+^. Anal. calcd for C_75_H_54_N_2_O_2_ (1014.4185).

## 3. Results and Discussion

### 3.1. Photophysical Properties

**Scheme 1:**
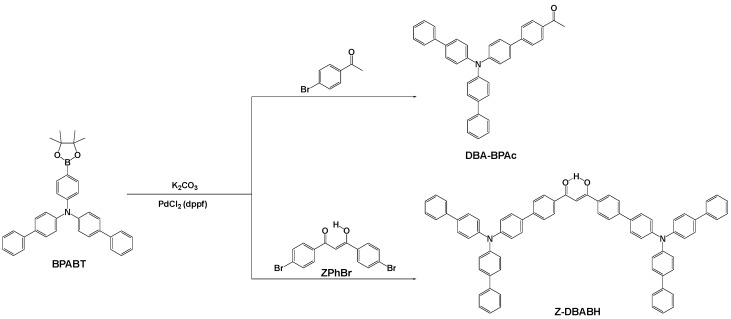
Synthetic Reaction scheme of DBA-BPAc and Z-DBABH.

**Figure 1:**
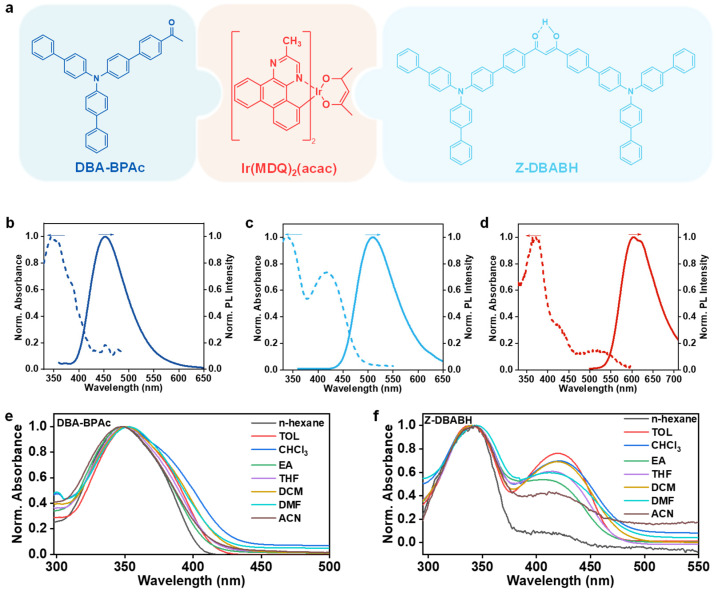
(**a**) Chemical structures of the two blue fluorophores and one red phosphorescent material. (**b**–**d**) The absorption and emission spectra of (**b**) DBA-BPAc, (**c**) Z-DBABH, and (**d**) Ir(MDQ)_2_(acac). (**e**,**f**) Normalized absorption spectra of (**e**) DBA-BPAc and (**f**) Z-DBABH in various solvents (1 × 10^−5^ M) at room temperature.

The D-A and D-A-D type blue-emitting luminophores, DBA-BPAc and Z-DBABH (molecular structures shown in Figure 1a), were designed and synthesized via Suzuki coupling reactions (Scheme 1). Both emitters are constructed on tris(biphenyl)amine donor scaffold with conjugated carbonyl acceptor moiety. The D-A-D type material of Z-DBABH exhibited larger molecular rigidity caused by intramolecular hydrogen bonding in the 3-hydroxypropenone and is, therefore, favorable for improving its thermal stability.

The absorption and emission properties of DBA-BPAc and Z-DBABH films (1 wt%) doped in polymethyl methacrylate (PMMA) matrices were investigated. As shown in Figure 1b, the DBA-BPAc film exhibited a distinct absorption band at 347 nm with a shoulder peak at 385 nm and a photoluminescence (PL) emission peaked at 454 nm, corresponding to a blue emission. In comparison, Z-DBABH displayed two absorption bands centered at 340 nm and 418 nm (Figure 1c). For both compounds, the shorter and longer-wavelength absorptions are attributed to the π → π* transition in the conjugated system and intramolecular charge transfer (ICT), respectively, which is consistent with the solvent dependence of the absorption spectra (Figure 1e,f). Moreover, Z-DBABH exhibited a significantly red-shifted PL emission which peaked at 510 nm relative to DBA-BPAc. This pronounced bathochromic shift is attributed to the more extended π-conjugation in Z-DBABH.

### 3.2. Factors for Designing High-Performance Film Thermometers

#### 3.2.1. Discrepancy in Thermal Decay of Fluorescence

**Figure 2:**
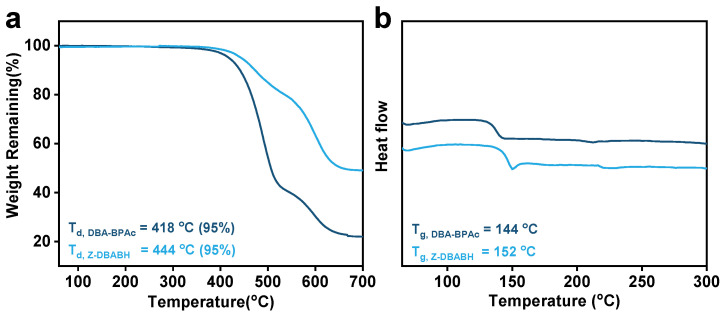
(**a**) TGA and (**b**) DSC results of DBA-BPAc and Z-DBABH.

To test the applicability in a high-temperature region, the thermal degradation temperatures of DBA-BPAc and Z-DBABH were measured, which were determined to be 418 and 444 °C, respectively (Figure 2a). Their glass transition temperatures were detected as 144 and 152 °C, respectively (Figure 2b). The superior thermal stability of material Z-DBABH is likely attributed to its more rigid and extended conjugated structure. The desirable thermal stability of both emitters ensures that their films can operate stably under high-temperature conditions.

**Figure 3:**
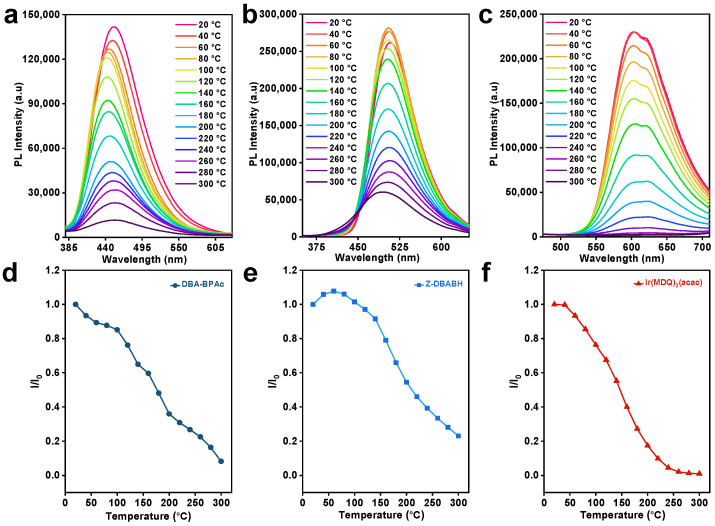
(**a**–**c**) Temperature-dependent emission spectra of (**a**) DBA-BPAc, (**b**) Z-DBABH, and (**c**) Ir(MDQ)_2_(acac). (**d**–**f**) The decay of the relative fluorescence intensities of the three emitters at heating.

Next, the thermal responsiveness of DBA-BPAc and Z-DBABH fluorescence in PMMA films (1 wt%) has been investigated in the temperature range of 20 to 300 °C (Figure 3a,b). To quantitatively evaluate their thermal quenching behaviors, the decay rates of the relative intensity change (ΔI/I_0_) are determined by the corresponding temperature interval (ΔT) (Figure 3d,e). As the temperature increased, the PL intensity of DBA-BPAc continuously declined throughout the temperature range of 20–300 °C (Figure 3a). In the range of 20–100 °C, DBA-BPAc exhibited an average fluorescence deactivation rate of 0.19% °C^−1^. At higher temperatures (100–300 °C), the decay rate markedly accelerated to 0.38% °C^−1^. Ultimately, the emission intensity decreased to 8.2% of its initial intensity at 300 °C (Figure 3d). In contrast, Z-DBABH showed a slight enhancement, reaching about 106% of its original intensity at 60 °C, followed by a continuous decrease at higher temperatures (Figure 3b,d). The abnormal increase in the emission intensity at heating is possibly caused by thermally populated high-level excited states. A slow average deactivation speed of 0.20% °C^−1^ was observed between 60 and 140 °C, which then accelerated to 0.43% °C^−1^ in the range of 140–300 °C, and the emission intensity dropped to 23.1% of its initial value at 300 °C.

To construct a ratiometric temperature-sensing system, a red-emitting phosphorescent material exhibiting a faster thermal response was introduced as a reference emitter. The Ir(MDQ)_2_(acac) film (1 wt% in PMMA polymer matrix) displayed an absorption band centered at 364 nm and a strong red emission peak at 605 nm with a shoulder at 621 nm (Figure 1d). As shown in the temperature-dependent PL spectra, the emission intensity of Ir(MDQ)_2_(acac) decreased continuously to 67.48% of its initial value at 120 °C, 9.79% at 240 °C, and eventually dropped to 0.9% at 300 °C (Figure 3c,d). In the range of 20–220 °C, the average fluorescence deactivation rate was 0.45% °C^−1^. When the temperature further rose to 220–300 °C, the decay rate slowed down to 0.11% °C^−1^, as Ir(MDQ)_2_(acac) had nearly complete thermal quenching, leading to an almost complete emission quenching at 300 °C. This difference in thermal response between the red and blue emitters provided a reliable foundation for realizing temperature sensing through ratiometric intensity measurements.

#### 3.2.2. Frustration of Energy Transfer

**Figure 4:**
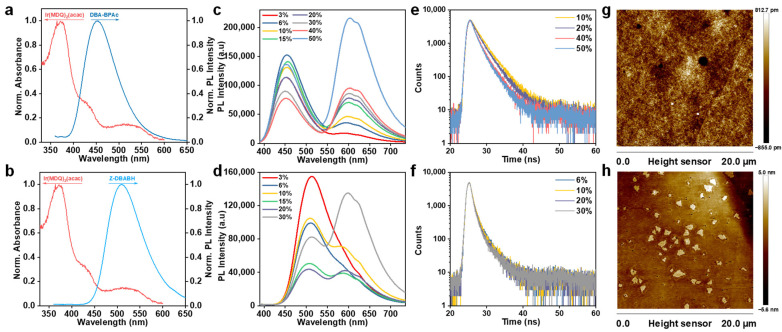
(**a**,**b**) Absorption spectrum of Ir(MDQ)_2_(acac) and PL spectra of (**a**) DBA-BPAc and (**b**) Z-DBABH. (**c**,**d**) Steady PL (excited at 380 nm) spectra of the (**c**) DBA-BPAc/Ir(MDQ)_2_(acac) and (**d**) Z-DBABH/Ir(MDQ)_2_(acac) hybrid films with different mixing ratios. (**e**,**f**) PL decay profiles of the (**e**) DBA-BPAc/Ir(MDQ)_2_(acac) and (**f**) Z-DBABH/Ir(MDQ)_2_(acac) hybrid films with different mixing ratios. (**g**,**h**) AFM topographic images of the (**g**) DBA-BPAc/Ir(MDQ)2(acac) film (DBA-BPAc: Ir(MDQ)_2_(acac) = 2:1 by weight, total dopant concentration = 1 wt%) and (**h**) Z-DBABH/Ir(MDQ)_2_(acac) film (Z-DBABH: Ir(MDQ)_2_(acac) = 10:3 by weight, total dopant concentration = 1 wt%).

Hybrid films were fabricated by incorporating the thermally stable blue emitters DBA-BPAc and Z-DBABH with the red phosphor Ir(MDQ)_2_(acac) into a PMMA matrix at various doping ratios, respectively. The spectra overlap between the emission spectra of both the two blue emitters and the absorption spectrum of Ir(MDQ)_2_(acac) is relatively small (Figure 4a,b). This suggests that the energy transfer in the hybrid films may be largely suppressed, which is vital for achieving high-performance thermosensing.

Further, the steady PL spectrum and fluorescence decay profiles at different mixing ratios were investigated. For the DBA-BPAc/Ir(MDQ)_2_(acac) hybrid films, a series of samples with Ir(MDQ)_2_(acac) contents of 3%, 6%, 10%, 15%, 20%, 30%, 40%, and 50% (relative to DBA-BPAc) were prepared (Figure 4c). Noted that even at the highest mixing ratio of Ir(MDQ)_2_(acac) (50%), the PL spectra still exhibited a distinct blue emission and the intensity ratio of blue to red emission remained approximately 1:2 (Figure 4c). Similarly, in the Z-DBABH/Ir(MDQ)_2_(acac) hybrid films, noticeable blue emission could still be detected even when the Ir(MDQ)_2_(acac) content reached 30%, with the blue-to-red intensity ratio at about 3:5 (Figure 4d). Such coexistence of strong dual emission with such high low-energy emitter concentrations is rarely observed in conventional energy-transfer systems, suggesting that the energy transfer in the hybrid films has been effectively inhibited.

Moreover, for the DBA-BPAc/Ir(MDQ)_2_(acac) hybrids, when the Ir(MDQ)_2_(acac) content increased from 10% to 50%, the fluorescence lifetime of DBA-BPAc detected at 480 nm decreased only slightly from 2.74 ns to 2.03 ns (Figure 4e and Table 1). In the Z-DBABH/ Ir(MDQ)_2_(acac) hybrid films, as the Ir(MDQ)_2_(acac) concentration increased from 6% to 30%, the Z-DBABH lifetime decreased marginally from 1.46 ns to 1.22 ns (Figure 4f and Table 2). Since ${\;\tau }_{D,A\;}$ only exhibits a slight decrease with increase in doping ratio of Ir(MDQ)2(acac), the efficiency of energy transfer (Φ_ET_) is not significantly affected, according to the equation of

(1) $$\Phi_{ET}=1-\frac{\tau_{D,A}}{\tau_{D,0}}$$

where $\tau_{D,0}$ and $\tau_{D,A}$ are the lifetimes in absence and with presence of acceptor, respectively. Such minimal lifetime changes further indicate that energy transfer from the blue donors to the red acceptor is largely suppressed. Additionally, the Z-DBA-BPAc/Ir(MDQ)_2_(acac) film obviously demonstrates significantly more pronounced phase separation compared to the DBABH/Ir(MDQ)_2_(acac) film (Figure 4g,h), which correlates well with its even lower energy transfer efficiency. The Z-DBABH/Ir(MDQ)_2_(acac) films clearly presents a large number of micron-to-nanoscale flake-like domains, with protrusion heights of 2–5 nm and lateral sizes ranging from 340 to 1820 nm. In contrast, the DBA-BPAc/Ir(MDQ)_2_(acac) films shows very few of such micro/nanoflake structures. Therefore, all these results revealed that the energy transfer from DBA-BPAc and Z-DBABH to Ir(MDQ)_2_(acac) is significantly inhibited, which is advantageous for achieving high-performance thermosensing abilities.

#### 3.2.3. Separation of Ratiometric Emissions

The difference in the emission peak wavelengths between DBA-BPAc and Ir(MDQ)_2_(acac) is measured as 151 nm, significantly greater than that between Z-DBABH and Ir(MDQ)_2_(acac) (95 nm). Consistently, the integrated area of the spectrum overlap between the normalized emission spectra of DBA-BPAc and Ir(MDQ)_2_(acac) is calculated to be 4.98, which is notably smaller than that between Z-DBABH and Ir(MDQ)_2_(acac), with a value of 18.12. The greater the distance between the emission peaks of the two emitters, the more effectively interference of the two emissions can be avoided, which helps to preserve their distinct thermal responsiveness in luminescent intensity. This can be a crucial factor for achieving high-performance ratiometric thermometers.

### 3.3. Ratiometric Thermosensing Performance of the Hybrid Films

We further investigated the temperature-dependent photoluminescence behavior of the energy-transfer-inhibited hybrid films. For these studies, the hybrid films were prepared in a PMMA matrix with a total dopant concentration of 1 wt%. The doping ratios were selected as 2:1 for DBA-BPAc/Ir(MDQ)_2_(acac) film and 10:3 for Z-DBABH/Ir(MDQ)_2_(acac) film, ensuring both blue and red emissions could be observed and enabling ratiometric thermal sensing.

**Figure 5:**
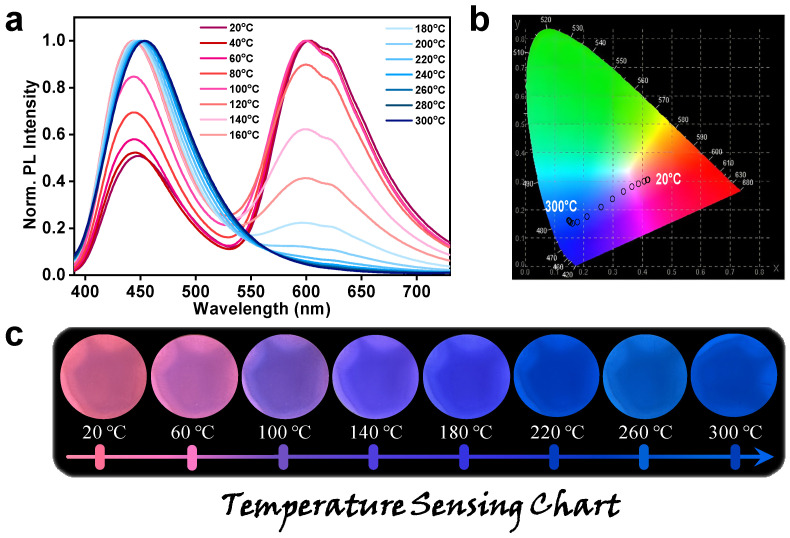
(**a**) Temperature-dependent emission spectra (excited at 380 nm) and (**b**) corresponding CIE coordinates. (**c**) Fluorescent photographs of the DBA-BPAc/Ir(MDQ)_2_(acac) (2:1) hybrid film at different temperatures.

For DBA-BPAc/Ir(MDQ)_2_(acac) hybrids, the hybrid film exhibits two distinct emission bands at 448 nm and 605 nm, corresponding to DBA-BPAc and Ir(MDQ)_2_(acac), respectively (Figure 5a). With increasing temperature, the blue emission attenuates more slowly than the red emission due to its higher thermal stability, and at the meantime, the blue peak exhibited a slight red shift from 448 nm to 454 nm. Upon heating from 20 to 140 °C, the emission is dominated by the red emission, whereas above 140 °C, the blue emission becomes dominant. Beyond 240 °C, the red emission peak nearly vanishes. During the entire heating process, the fluorescence color of the hybrid film gradually changes from red to pink, then to purple, violet, and finally deep blue, with the corresponding Commission Internationale de l’Éclairage (CIE) coordinates shifting progressively from (0.43, 0.30) at 20 °C to (0.40, 0.29) at 80 °C, (0.31, 0.24) at 140 °C, (0.22, 0.17) at 180 °C, and eventually to (0.16, 0.16) at 300 °C (Figure 5b,c), confirming the naked-eye visual detectability of the film thermometer.

**Figure 6:**
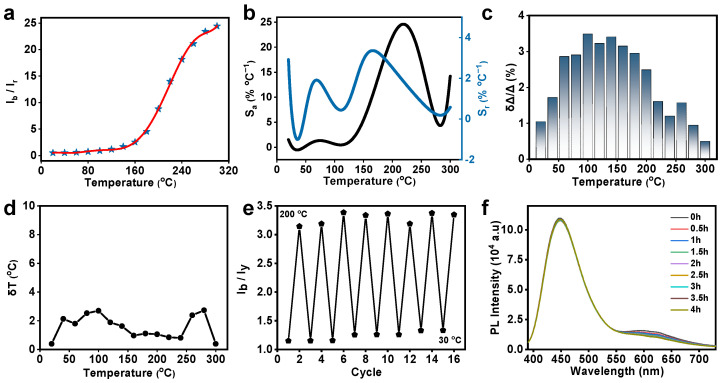
(**a**) Temperature dependence of the fluorescence intensity ratio of the blue to red emission. (**b**) Relative and absolute sensitivity, (**c**) relative error, (**d**) temperature uncertainties, (**e**) fluorescence ratio intensity of hybrid film upon cycling the temperature between 30 and 200 °C, and (**f**) the emission spectra of the film measured at 200 °C during 4 h of heating.

The fluorescence intensity ratio I_b_/I_r_ exhibits a monotonic dependence on temperature (Figure 6a), which can be quantitatively described by the following seventh-order polynomial function of temperature T (°C):

Y = −1.09014 + 0.18573 T − 0.00773 T^2^ + 1.51917 × 10^−4^ T^3^ −1.52888 × 10^−6^ T^4^ + 8.04697 × 10^−9^ T^5^ − 2.06886 × 10^−11^ T^6^ + 2.04658 × 10^−14^ T^7^. (2)

This function provided an accurate representation of the ratiometric fluorescence changes over the measured temperature range. The absolute sensitivity (S_a_) of the hybrid film remains higher than 1.0% °C^−1^ across the temperature range of 124–300 °C, reaching a maximum of 24.61% °C^−1^ at 219 °C (Figure 6b). The relative sensitivity (S_r_) exceeds 0.43% °C^−1^ over a broader range of 50–265 °C, with higher values above 1.0% °C^−1^ observed within narrower temperature windows of 54–92 °C and 126–245 °C, achieving a peak value of 3.36% °C^−1^ at 166 °C. The relative error remains below 3.48% throughout the entire temperature range (20–300 °C), reaching a minimum of 0.49% at 300 °C (Figure 6c). Moreover, the temperature resolution is below 2.7 °C across the full temperature window (Figure 6d). Within the high-temperature range of 120–240 °C, the temperature resolution improves further to below 2.0 °C, reaching a best performance of 0.81 °C at 240 °C (Figure 6d). Notably, this region coincides with the temperature range where S_r_ > 1% °C^−1^ (126–245 °C), confirming that the ratiometric film thermometer is reliable and highly sensitive in this temperature window. Furthermore, the sensing film exhibits excellent reversibility and thermal stability, with the I_b_/I_r_ ratios remaining highly repeatable upon multiple heating–cooling cycles between 30 °C and 200 °C and exhibiting no significant change upon continuous heating at 200 °C for 4 h (Figure 6e,f).

**Figure 7:**
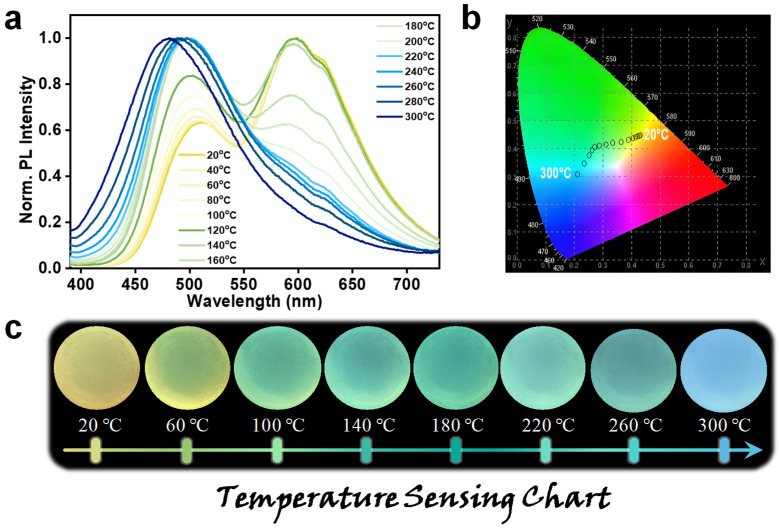
(**a**) Temperature-dependent emission spectra (excited at 380 nm) and (**b**) corresponding CIE coordinates. (**c**) Fluorescent photographs of the Z-DBABH/Ir(MDQ)_2_(acac) (10:3) hybrid film at different temperatures.

**Figure 8:**
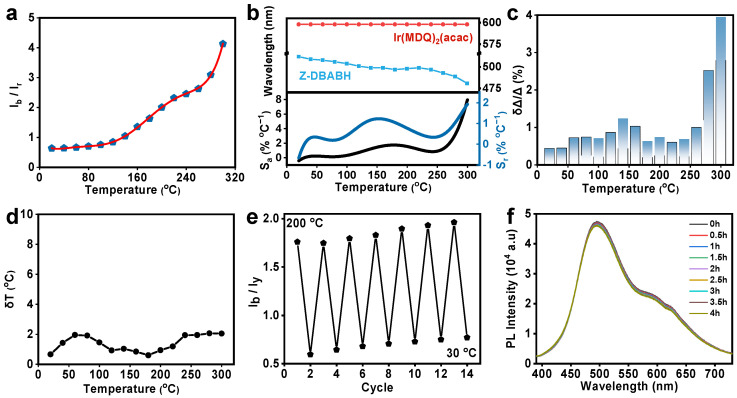
(**a**) Temperature dependence of the fluorescence intensity ratio of the blue-to-red emission. (**b**) Temperature dependence of the emission wavelength for Z-DBABH and Ir(MDQ)_2_(acac), relative and absolute sensitivity, (**c**) relative error, (**d**) temperature uncertainties, (**e**) fluorescence ratio intensity of hybrid film upon cycling the temperature between 30 and 200 °C, and (**f**) the emission spectra of the film measured at 200 °C during 4 h of heating.

For the Z-DBABH/Ir(MDQ)_2_(acac) hybrid films, the blue and red emission peaks are located at 512 nm and 598 nm at room temperature, respectively (Figure 7a). The fluorescence intensity ratio I_b_/I_r_ increases gradually between 20 and 140 °C and rises more rapidly from 140 to 300 °C, indicating an accelerated thermal quenching of the red-emitting Ir(MDQ)_2_(acac) component above 140 °C (Figure 8a). Correspondingly, the CIE coordinates shift from (0.45, 0.45) at 20 °C to (0.43, 0.44) at 80 °C, (0.33, 0.41) at 180 °C, and finally to (0.23, 0.31) at 300 °C, indicating a gradual color transition from yellow to green, cyan, and ultimately blue, indicating visually perceptible temperature detection ability (Figure 7b,c).

The temperature-dependent fluorescence intensity ratio I_b_/I_r_ was fitted using the following seventh-order polynomial:

Y = 0.92122 − 0.02803 T + 9.40319 × 10^−4^ T^2^ − 1.41819 × 10^−5^ T^3^ + 1.07669 × 10^−7^ T^4^ − 3.976 × 10^−10^ T^5^ + 6.49418 × 10^−13^ T^6^ − 3.17656 × 10^−16^ T^7^ (3)

with an excellent correlation coefficient of R^2^ = 0.9996 (Figure 8a). The Z-DBABH/Ir(MDQ)_2_(acac) hybrid film shows a reliable but inferior sensing performance that the DBA-BPAc/Ir(MDQ)_2_(acac) film. The S_a_ remained above 0.83% °C^−1^ throughout the temperature range of 123–300 °C and reached a maximum value of 7.96% °C^−1^ at 300 °C (Figure 8b). The S_r_ was higher than 0.33% °C^−1^ within the wide range of 93–300 °C, exceeding 1.0% °C^−1^ in two narrower temperature windows of 127–183 °C and 278–300 °C, and showing a maximum of 1.92% °C^−1^ at 300 °C (Figure 8b). The relative error remained below 2.0% in the 20–260 °C range and increased slightly to 2.52% at 280 °C and 3.95% at 300 °C, respectively, demonstrating good measurement reliability (Figure 8c). The temperature resolution was below 2.06 °C across the entire range and achieved the best value of 0.60 °C at 180 °C, indicating excellent temperature discrimination capability (Figure 8d). Compared with the DBA-BPAc/Ir(MDQ)_2_(acac), the thermal cycling stability was slightly inferior and exhibited minor fluctuations within a certain range (Figure 8e). Nevertheless, the hybrid film still demonstrated excellent thermal durability, showing no significant degrade after heating at 200 °C for 4 h (Figure 8f). These ratiometric film thermometers demonstrate excellent sensing performance in the high-temperature region, ranking among the best of high-temperature ratiometric thermal sensors constructed from either organic or inorganic materials (Table 3) [34,35,36,37,40,41,42,43].

### 3.4. Analysis of Determinant Factors for Themosensing

From the previous discussion, three key factors have been demonstrated to influence ratiometric thermosensing performance: the discrepancy in fluorescence thermal decay, the frustration of energy transfer, and the separation of ratiometric emissions. Among the two hybrid systems, the Z-DBABH/Ir(MDQ)_2_(acac) film exhibits a larger thermal decay discrepancy and lower energy transfer efficiency. However, despite its display of reliable sensing capability, this system still shows lower sensitivity compared with the DBA-BPAc/Ir(MDQ)_2_(acac) hybrids. This finding indicated that the enlargement of the emission spectral separation plays a more decisive role in determining sensitivity than the other two factors.

Moreover, in the Z-DBABH/Ir(MDQ)_2_(acac) system, the maximum emission peak of Z-DBABH gradually blue-shifts from 512 nm to 497 nm upon heating from 20 to 180 °C, thereby increasing its spectral separation from Ir(MDQ)_2_(acac) (Figure 7a and Figure 8b). In this temperature window, both S_a_ and S_r_ exhibited an overall increasing trend (Figure 8b). The blue emission slightly red-shifts to 499 nm from 180 °C to 220 °C and then blue-shifts again to 481 nm from 220 °C to 300 °C, corresponding to a decrease and increase in sensitivity, respectively. This, with increased ET efficiency caused by hypochromism of blue emission and unchanged discrepancy in luminescence thermal decay, further demonstrates that the separation of the two emissions is more vital for enhancing the thermometry sensitivity.

## 4. Conclusions

In conclusion, this study highlight that achieving high-sensitivity ratiometric thermosensing at elevated temperatures requires a large difference in thermal responsiveness of fluorescence, minimizing spectral overlap of ET, and more importantly, enlargement of emission spectral separation. Benefiting from the larger emission spectral separation, the DBA-BPAc/Ir(MDQ)_2_(acac) film exhibits superior sensing performance, achieving a maximum S_r_ and S_a_ of 3.36% °C^−1^ (166 °C) and 24.61% °C^−1^ (219 °C), respectively, maintaining an S_r_ above 0.43% °C^−1^ in the range of 50–265 °C. In contrast, the Z-DBABH/Ir(MDQ)_2_(acac) film displays reliable but lower sensitivity, with an S_r_ reaching maximum of 1.92% °C^−1^ (300 °C), and above 0.33% °C^−1^ in range of 93–300 °C, yet offering superior temperature resolution, with values below 2.06 °C across the entire range. Moreover, both hybrid films exhibit distinct color transitions upon heating, indicating promising potential for multi-level optical anti-counterfeiting applications. Overall, these findings providing new insights for the design of high-performance ratiometric film thermometers.

## Figures and Tables

**Table 1:** Fitting data of the fluorescence decay of the DBA-BPAc/Ir(MDQ)_2_(acac) film (in the PMMA matrix) at different temperatures, detected at 480 nm.

Ratio	τ_1_/ns	τ_2_/ns	<τ> ^1^/ns	ꭓ^2^
10%	2.00 (54.60%)	3.63 (45.40%)	2.74	1.220
20%	1.68 (50.25%)	3.32 (49.75%)	2.50	1.269
40%	1.28 (48.19%)	2.85 (51.81%)	2.09	1.232
50%	1.19 (47.15%)	2.78 (52.85%)	2.03	1.287

^1^ The average lifetime.

**Table 2:** Fitting data of the fluorescence decay of the Z-DBABH/Ir(MDQ)_2_(acac) film (in the PMMA matrix) at different temperatures, detected at 480 nm.

Ratio	τ_1_/ns	τ_2_/ns	<τ> ^1^/ns	ꭓ^2^
6%	0.72 (63.62%)	2.76 (36.38%)	1.46	1.311
10%	0.75 (67.18%)	2.87 (32.82%)	1.45	1.255
20%	0.71 (70.58%)	2.78 (29.42%)	1.32	1.212
30%	0.66 (73.33%)	2.74 (26.67%)	1.22	1.347

^1^ The average lifetime.

**Table 3:** Comparison of sensing performance with other inorganic and organic ratiometric thermometers.

Material	Eye- Detectable	Maximum S_r_	Temperature Range with S_r_ > 0.5% °C^−1^	Temperature range with S_r_ > 1% °C^−1^	Recyclability	Film Thermal Sensor	Fabrication Method	Ref.
DBA-BPAc: Ir(MDQ)_2_(acac)	Yes	3.36% °C^−1^ (at 166 °C)	50–105 °C, 116–262 °C	54–92 °C, 126–245 °C	√ (20–200 °C)	√	drop-casting	This work
Z-DBABH: Ir(MDQ)_2_(acac)	Yes	1.92% °C^−1^ (at 300 °C)	103–220 °C, 261–300 °C	127–183 °C, 278–300 °C	√ (20–200 °C)	√	drop-casting	This work
S1/C2	Yes	2.49% °C^−1^ (at 220 °C)	40–300 °C	120–300 °C	√	√	drop-casting	Ref. [36]
P1/CzLA	Yes	1.12% °C^−1^ (at 156 °C)	102–236 °C	133–180 °C	√	√	drop-casting	Ref. [37]
C3/T4AC	Yes	1.27% °C^−1^ (at 128 °C)	90–189 °C	− ^1^	√	√	drop-casting	Ref. [35]
P1/T4AC	Yes	2.14% °C^−1^ (at 168 °C)	23–247 °C	50–225 °C	√	√	drop-casting	Ref. [40]
C2/HBTPy	Yes	6.32% °C^−1^ (at 167 °C)	52−170 °C	90–170 °C	√	√	drop-casting	Ref. [34]
BaTiO_3_: Yb^3+^: Tm^3+^: Er^3+^	− ^1^	2.70% °C^−1^ (at 30 °C)	30−120 °C 130–270 °C	30−120 °C 130–270 °C	− ^1^	×	− ^1^	Ref. [41]
Dy^3+^-doped GdVO_4_	− ^1^	2% °C^−1^ (at 27 °C)	27–200 °C	27–157 °C	√	√	pulsed laser deposition	Ref. [42]
YVO_4_: Eu^3+^: Dy^3+^	Yes	3.6% °C^−1^ (at 367 °C)	277–427 °C	277–427 °C	− ^1^	√	pulsed laser deposition	Ref. [43]

^1^ not available.

## Data Availability

The data will be made available upon reasonable request.
